# Revista Brasileira de Medicina do Trabalho is accepted in SciELO

**DOI:** 10.47626/1679-4435-2023-214

**Published:** 2024-02-16

**Authors:** Andrea Franco Amoras Magalhães, Mírian Perpétua Palha Dias Parente

**Affiliations:** Editors-in-Chief of Revista Brasileira de Medicina do Trabalho

We are concluding 2023 with the great news of our admission to SciELO. For many years, we have been striving for inclusion in this important database and, finally our efforts have paid off. We extend our gratitude for the guidance and support throughout the evaluation process to everyone who contributed to this achievement: the Board of Associação Brasileira de Medicina do Trabalho (ANAMT), the Editorial Board and contributors of Revista Brasileira de Medicina do Trabalho (RBMT), reviewers, authors, and the SciELO team.

It is important to note that inclusion in SciELO is only the first step. For our permanence in the database, we will need to continue adhering to a set of standards, called the "SciELO criteria," which will require even greater efforts from everyone involved in the production and editorial teams of RBMT In addition to this major achievement, we would also like to share some of RBMT's actions over the past year.

In December 2022, RBMT started publishing articles ahead of print (AOP), which is the early or advanced publication of peer-reviewed articles. This approach expedites article availability, considering the large number of manuscripts RBMT receives, which often leads to a substantial period between approval and publication in an issue. Since then, 125 articles have been made available AOR The main advantage is that once available, the articles can be read and cited in other studies, potentially increasing both the journal's and authors' citation counts.

In June of this year, RBMT was accepted into the Directory of Open Access Journals (DOAJ), an important database that will increase the journal's visibility, allowing its articles to be more cited and, consequently, attracting more authors to publish with us. Additionally, acceptance into DOAJ was one of the prerequisites for indexing in SciELO.

Also in 2023, the journal adopted a continuous article publication model. The primary objective was to expedite article availability and, similar to AOP, contribute to their reading and citation.

Improvements were made to the Author Guidelines according to the SciELO criteria, including information such as support for open science, acceptance of preprints, anti-plagiarism policy incorporation of a section on the use of artificial intelligence in articles, among others.

Another improvement implemented was the mandatory inclusion of the Open Researcher and Contributor ID (ORCID) for all co-authors of an article. ORCID is a unique identifier assigned to each author, linking them to their publications, thus facilitating scientific communication and dissemination. Consequently, it is essential for authors to be identified, ensuring their publications are easily found.

The year 2023 was marked by various challenges, but RBMT remained committed to its mission of publishing original articles while improving editorial practices. In 2024, we will continue refining and improving the editorial process, applying all efforts and actions to maintain our journal indexed in SciELO, which will make us rise to the standard of major journals.

On behalf of the Scientific Board and the editorial team of RBMT, we wish everyone peace, health, success, and a happy 2024!

